# Effects of Different Pressure Levels in Topical Negative Pressure Application—Analysis of Perfusion Parameters in a Clinical Skin Model Using Multimodal Imaging Techniques

**DOI:** 10.3390/jcm11175133

**Published:** 2022-08-31

**Authors:** Emine Ceylan Aslan-Horch, Raymund E. Horch, Andreas Arkudas, Wibke Müller-Seubert, Ingo Ludolph

**Affiliations:** Department of Plastic and Hand Surgery and Laboratory for Tissue Engineering and Regenerative Medicine, University Hospital Erlangen, Friedrich-Alexander-Universität Erlangen-Nürnberg (FAU), Krankenhausstraße 12, 91054 Erlangen, Germany

**Keywords:** negative pressure wound therapy, perfusion, pressure level, imaging, thermography, near infrared spectroscopy

## Abstract

The effects of topical negative pressure therapy (TNP) have been a subject of research for many years. In this study, we investigated new imaging devices to detect clinical changes that TNP causes on healthy tissue and identified differences in microcirculation created by different pressure levels. We used near-infrared spectroscopy (NIS), thermography, and a vein illuminator to measure the differences in oxygen saturation, tissue temperature, and vein pattern. A control group (−125 mmHg) and three comparison groups with only TNP dressing (Group 1), −25 mmHg (Group 2), and −175 mmHg (Group 3) were established. Thirty minutes of TNP on intact skin was followed by 30 min of resting. A total of 24 participants were measured by all imaging devices at predetermined time points. Oxygen saturation and skin temperature increased by 8.07% and 1.67 °C for the control group, 4.00% and 1.65 °C for Group 2, and 8.45% and 1.68 °C for Group 3. Group 1 showed a slight increase in oxygen saturation and a 2.7 °C increase in skin temperature. Over the 30 min following removal of TNP, oxygen saturation and temperature decreased gradually for all groups. The vein illuminator did not show significant differences in the venous pattern or flow. Our study showed that higher negative pressure values resulted in higher oxygen saturation and higher tissue temperature.

## 1. Introduction

In modern wound treatment, negative pressure wound therapy (NPWT) has evolved to be a standard option, especially for wound bed preparation. It is widely used for different indications such as chronic or infected wounds or as a temporary wound closure for defect coverage following trauma or tumor operations, and it is increasingly applied in the form of vacuum wound therapy with instillation [1,2,3,4,5]. Therefore, indications have significantly broadened since the first introduction of NPWT devices, and topical negative pressure has been advocated over closed incisions to prevent surgical site infections [6,7,8].

The debate about the exact mechanisms of NPWT is still ongoing, although different effects have been well assessed. Especially, the influence of NPWT on wound bed or tissue perfusion regarding the subatmospheric pressure used is not yet fully understood.

In the late 1990s, Morykwas et al. presented the first results of vacuum-assisted wound therapy, which reported an increase in tissue perfusion using NPWT in a swine model. Moreover, they assessed different levels of negative pressure in their model and observed favorable results using a subatmospheric pressure of −125 mmHg. Since then, a plethora of studies have investigated different effects of NPWT in the context of tissue perfusion, pressure distribution, or bacterial burden, as well as in the application of various wound situations [9,10,11].

Recent developments in technologies used for the analysis of tissue perfusion provided motivation to assess different pressure levels of NPWT in a clinical skin model. Both oxygen saturation and tissue temperature are important parameters in wound healing. The involvement of the parameters is undisputable but not fully understood for the various processes involved [12,13,14,15]. As previously published, near-infrared spectroscopy and thermography are able to detect changes in skin perfusion following NPWT [16]. In addition to those technologies, our results present venous perfusion analysis acquired from a laser-driven device. We hypothesized that higher levels of negative pressure would result in lower levels of tissue oxygen at the end point of 30 min topical negative pressure.

## 2. Material and Methods

A total of 27 healthy subjects participated in the study and provided written informed consent. Three female participants were not able to complete the study for technical reasons and were therefore excluded from the data analysis. Participants reported no relevant comorbidities and were randomly assigned to the different groups while keeping the female-to-male ratio similar. A positive ethical vote from the institutional ethics committee is registered (registration number 310_19 B).

### 2.1. Blood Flow Measurement Devices

Near-infrared spectroscopy (Snapshot NIR^®^; KENT Imaging Inc., Calgary, AB, Canada) and thermographic imaging (FLIR ONE Pro, FLIR Systems, Inc., Wilsonville, OR, USA) were used for the measurement of the oxygenation and the temperature of the affected skin, respectively. The techniques have been explained in detail elsewhere [16,17].

As a new method for the evaluation of venous blood perfusion, a vein illuminator (AccuVein AV500, AccuVein Inc., Medford, NY, USA) was used to find out if additional information about the venous flow pattern could be gained. The device uses near-infrared (NIR) imaging and was originally developed to visualize blood vessels in order to support venipuncture. It emits infrared light with a wavelength of 785 nm. After capturing the reflected light from the participant’s body, the resulting image is projected onto the participant’s skin, creating a real-time reproduction of the subcutaneous vein pattern. Because infrared light is absorbed by the hemoglobin, blood-filled structures such as the veins appear as dark lines compared to the adjacent tissue that reflects the green light. The device offers an inverted mode where the veins are seen as luminous green lines on a dark background. The implemented lasers together provide an image of the subcutaneous vascular structure up to 10 mm depth.

**Figure 1:**
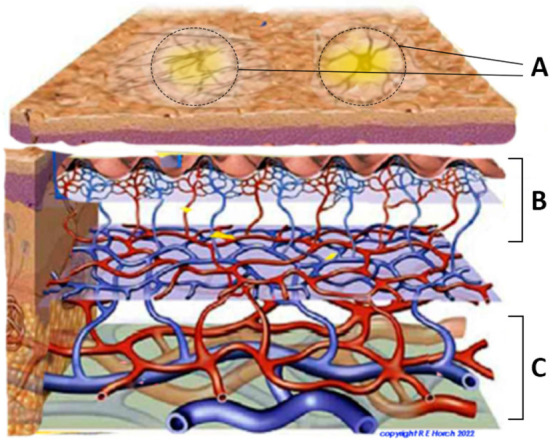
Potential projection of direct perforator vessels at the skin (spotted rings) as they could be detected by thermography as so-called hot spots. (**A**) “Hot spot” projection: circled areas show enhanced heat pattern over perforating vessels and can be visualized by thermography. (**B**) Subcutaneous perforator vessels branching into skin. (**C**) Axial submuscular blood vessels.

The different imaging modalities were applied to reflect various possible dimensions of tissue perfusion. In contrast to spectroscopy, thermography relies on thermal heat readings, which are supposed to represent an entire surface or regions of interests within it as well as the location of potential perforating blood vessels (Figure 1).

### 2.2. Topical Negative Pressure Application

Standardized distances and five identical regions of interests at the thigh of the participants with a single diameter of 9 mm were defined and applied to all participants in the same way for the near-infrared spectroscopy (NIS) and the thermography. Average values out of the regions of interest were calculated and used for further statistical analysis. We performed visual comparisons of the pictures taken from the vein finder. All measurements were done by the same person to ensure there were no differences in measuring techniques.

Study measurements were all done at room temperature. Participants were asked to undress, and the area was determined by measuring the middle point from the lateral superior patella to the anterior superior iliac spine (anterolateral thigh region). The area was carefully shaved to prevent any image disruptions. Participants were then asked to rest for 10 min. After resting, t0 images were captured. In total, 3 Groups were defined (Table 1). For every participant, independent of the group, the right leg was defined as control and −125 mmHg topical negative pressure application (TNPA) (3M, KCI—an Acelity company, San Antonio, TX, USA) was applied using a medium-sized sponge continuously for 30 min.

For the left leg at the same anatomical area, different pressure levels were used to compare the effects of different TNPA levels on the tissue oxygen saturation, skin temperature, and changes in vein imaging, while keeping the other settings of TNPA the same. For Group 1, the TNPA was put on the left leg, but no negative pressure was established, to see the effect caused by the dressing itself on the skin area. For group 2, −25 mmHg TNPA was applied, and for Group 3, −175 mmHg TNPA was applied. After the TNPA was removed, t1 (30 min) images were captured. Final images were captured 30 min following the removal of TNPA (t6). Participants stayed rested and were not allowed to move or cover the area throughout the study duration.

**Figure 2:**
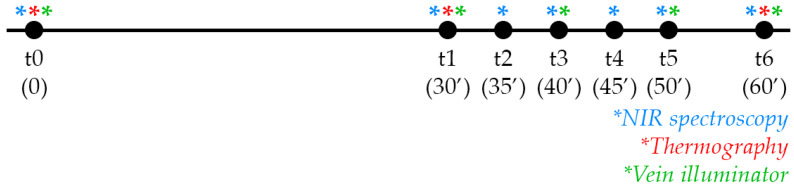
Timeline of the study. Different colors representing different devices used at certain timepoints.

For NIS, images were captured at different time intervals (measured in min) t0 (0’), t1 (30’), t2 (35’), t3 (40’), t4 (45’), t5 (50’), and t6 (60’) (Figure 2). The device was held approximately 30 cm away from the participant, following the guide of two central laser markers overlapping at the ideal distance for clear image capturing. Five regions of interest, corresponding with 5 laser points of the camera, were recorded for oxygen saturation, and the mean values for each image were calculated. 

For thermal imaging, images were captured at t0, t1, and t6. (Figure 2) The images were captured 50 cm away from the participant to capture the entire thigh area. The same 5 regions of interest as those used for spectroscopy were marked and calculated using an external software (FLIR Tools 6.4, FLIR Systems Inc., Wilsonville, Oregon, USA). The mean values from the 5 regions of each image were calculated and used in statistical calculations.

For the vein illuminator, images were captured 50 cm away from the object to cover the entire anterolateral thigh region. Images of the displayed vein patterns were captured using a Canon digital camera, as the device itself provides real-time images only and does not have a built-in camera for recording. Both devices were directed at the area from the same distance, and images were captured at t0, t1, t3, t5, and t6 (Figure 2). Because this device does not do objective measuring, quantitative data could not be gathered.

The Wilcoxon signed-rank test was used for the comparison of the nonparametric, related samples. Statistical analyses were compiled in IBM SPSS Statistics 23.0 (Armonk, NY, USA), and graphics were created using Microsoft Excel 2021 (Richmond, WA, USA).

## 3. Results

We investigated 16 male and 8 female participants with a mean age of 31.3 years (SD: 9.85; range: 23–62) and a mean body mass index (BMI) of 26 kg/m^2^ (SD: 6.81; range: 18.2–41.9).

### 3.1. Tissue Oxygen Saturation by NIS

**Figure 3:**
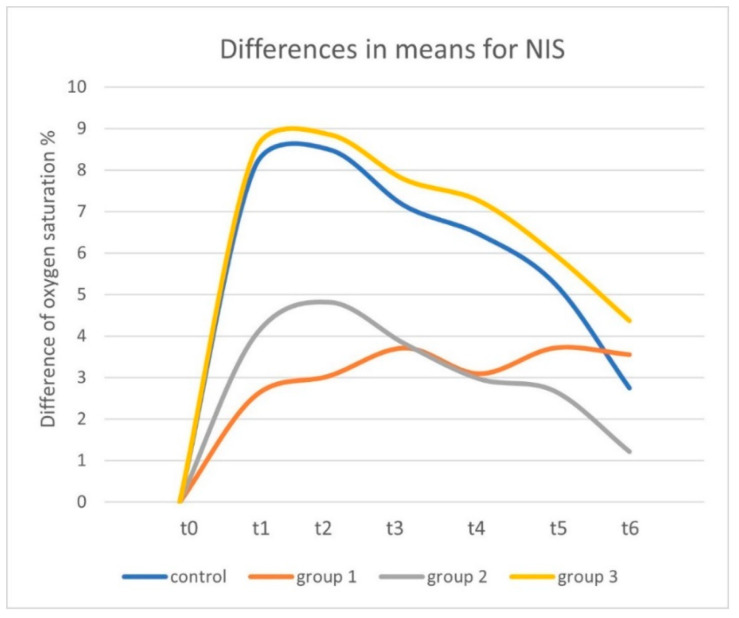
Differences of the means in the oxygen saturation compared to t0 over study timeline for near-infrared spectroscopy images.

**Figure 4:**
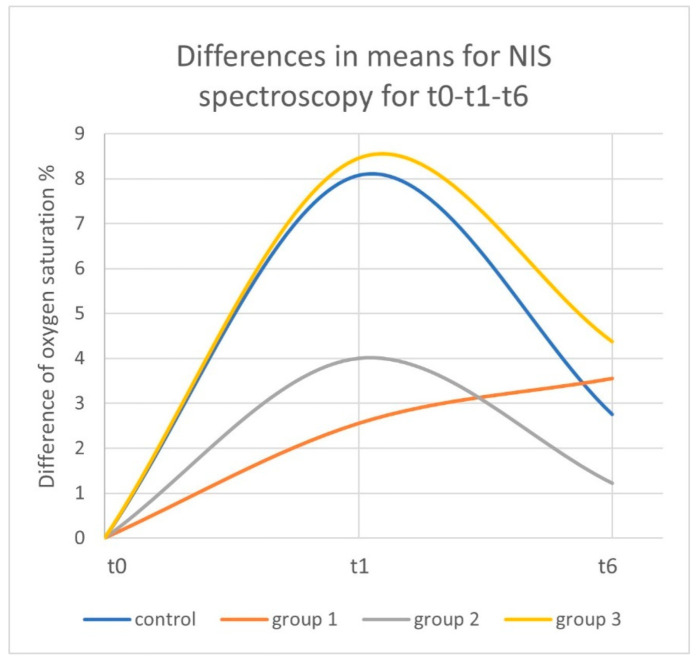
Differences of the means in the oxygen saturation percentage compared to t0 at t1 and t6 measured by near-infrared spectroscopy.

**Figure 5:**
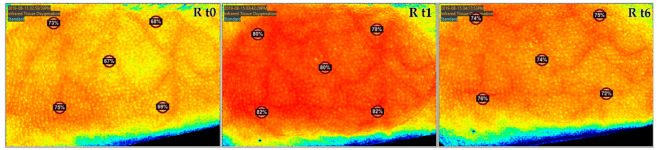
Near-infrared spectroscopy images from the control group, taken at t0 (**left**), t1 (**middle**), and t6 (**right**).

For the control group with the standard −125 mmHg negative pressure, tissue oxygen saturation showed an increase of 8.07% from 63.75% (SD: 11.41; range: 30.40–77.20) at t0 to 71.82% (SD: 8.34; range: 53.40–81.80) at t1 (*p* < 0.01). In the following 30 min after TNPA removal, oxygen saturation decreased by 5.32% to 66.50% (SD: 10.59; range: 47.20–79.60) at t6 (*p* < 0.01). Overall, a 2.75% increase was observed between t0 images and t6 (30 min after removal of TNPA) (*p* = 0.063) (Figure 3, Figure 4 and Figure 5) (Table 2).

For Group 1 without topical negative pressure, tissue oxygen saturation showed an increase of 2.55% from 65.22% at t0 (SD: 10.97; range: 39.60–77.20) to 67.77% at t1 (SD: 15.73; range: 29.80–79.80) (*p* ≤ 0.214). At t6, it reached 68.77% (SD: 9.41; range: 50.20–80.60) (*p* = 0.678). Overall, an increase of 3.55% was observed between t0 and t6 (*p* = 0.086) (Figure 3, Figure 4 and Figure 5) (Table 2).

For Group 2 with −25 mmHg negative pressure, tissue oxygen saturation showed an increase of 4.00% from 64.40% (SD: 13.24; range: 37.60–76.80) at t0 to 68.40% (SD: 11.94; range: 47.00–77.40) at t1 (*p* = 0.074). Thirty minutes after the removal of TNPA, oxygen saturation decreased to 65.62% (SD: 12.99; range: 41.40–77.80) at t6 (*p* = 0.28). Overall, an increase of 1.22% was observed between t0 and t6 (*p* = 0.395) (Figure 3, Figure 4 and Figure 5) (Table 2).

For Group 3 with −175 mmHg negative pressure, tissue oxygen saturation showed an increase of 8.45% from 61.45% (SD: 13.08; range: 33.40–74.00) at t0 to 69.90% (SD: 9.51; range: 56.80–83.40) at t1 (*p* = 0.012). Thirty minutes after the removal of TNPA, oxygen saturation decreased to 65.82% (SD: 9.36; range: 55.40–79.60) at t6 (*p* = 0.012). Overall, an increase of 4.37% was observed between t0 and t6 (*p* = 0.183) (Figure 3, Figure 4 and Figure 5). An overview of all results is given in Table 2.

### 3.2. Skin Temperature

**Figure 6:**
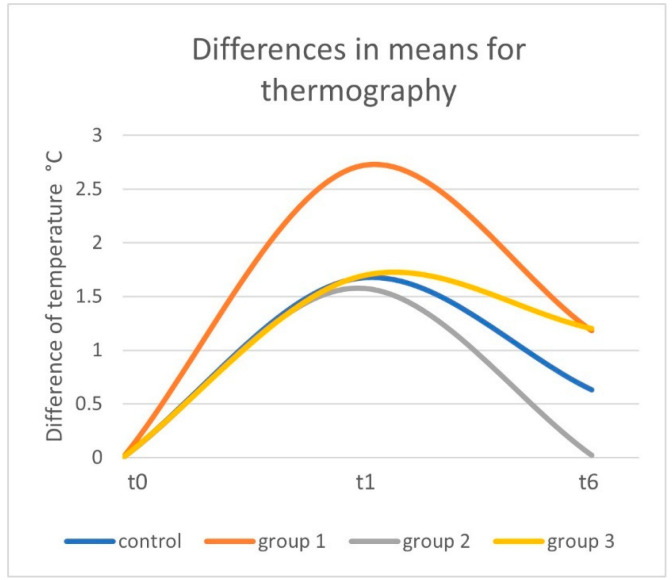
Differences of the means for the temperature in Celsius degrees compared to t0 at t1 and t6, measured by thermography.

For the control group, tissue temperature showed an increase of 1.67 °C from 30.60 °C (SD: 2.10; range: 25.10–34.5 °C) at t0 to 32.27 °C (SD: 2.06; range: 29.5–36.5 °C) at t1 (*p* < 0.01). In the following 30 min, the temperature decreased to 31.23 °C (SD: 2.24; range: 27.8–36.5 °C) at t6 (*p* < 0.01). Overall, an increase of 0.63 °C was observed between t0 and t6 (30 min after removal of TNPA) (*p* = 0.407) (Table 3) (Figure 6).

**Figure 7:**
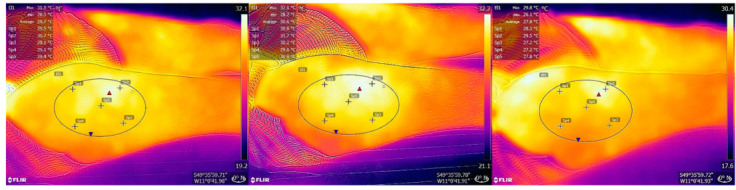
Thermography images from the control group, taken at t0 (**left**), t1 (**middle**), and t6 (**right**).

For Group 1, the tissue temperature showed an increase of 2.7 °C from 30.04 °C (SD: 2.23; range: 26.70–34.70 °C) at t0 to 32.75 °C (SD: 1.89; range: 30.7–36.0 °C) at t1 (*p* < 0.01). In the following 30 min, the temperature decreased to 31.22 °C (SD: 1.88; range: 29.2–34.9 °C) at t6 (*p* = 0.011). Overall, an increase of 1.18 °C increase was observed between t0 and t6 (*p* = 0.0154) (Table 3) (Figure 6 and Figure 7).

For Group 2 with −25 mmHg negative pressure, the tissue temperature showed an increase of 1.65 °C from 31.82 °C (SD: 1.67; range: 29.90–34.10 °C) at t0 to 33.4 °C (SD: 1.86; range: 31.3–36.2 °C) at t1 (*p* = 0.028). In the following 30 min, the temperature decreased to 31.84 °C (SD: 2.22; range: 28.7–34.6 °C) at t6 (*p* = 0.018). Overall, an increase of 0.02 °C was observed between t0 and t6 (*p* = 0.866) (Table 3) (Figure 6).

For Group 3 with the −175 mmHg negative pressure, the the tissue temperature showed an increase of 1.68 °C from 31.55 °C (SD: 1.41; range: 29.20–33.50 °C) at t0 to 33.23 °C (SD: 1.93; range: 31.0–36.10 °C) at t1 (*p* = 0.012). In the following 30 min, the temperature decreased to 32.75 °C (SD: 2.38; range: 30.0–35.8 °C) at t6 (*p* = 0.036). Overall, an increase of 1.2 °C was observed between t0 and t6 (*p* = 0.036) (Table 3) (Figure 6).

Table 3 provides an overview of all results of the skin temperatures for the different groups.

### 3.3. Vein Illuminator

**Figure 8:**
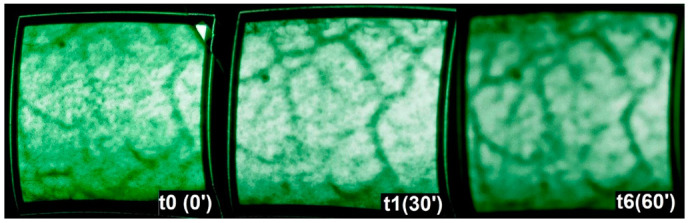
Vein illuminator images, showing patterns of superficial veins, captured by a digital camera at t0 (0’) (**left**), t1 (30’) (**middle**), and t6 (60’) (**right**).

Due to the design of the device, the vein illuminator is not capable of delivering quantitative measurements. It produces optical images of the venous blood flow pattern. The instrument creates live images, which were then captured using another digital camera. Comparing the images of the vein finder captured throughout the examination time, no significant differences could be detected between t0, t1, and t6 in the venous pattern, but the veins seemed to vary slightly in terms of thickness or vessel diameter, as shown in Figure 8.

## 4. Discussion

**Figure 9:**
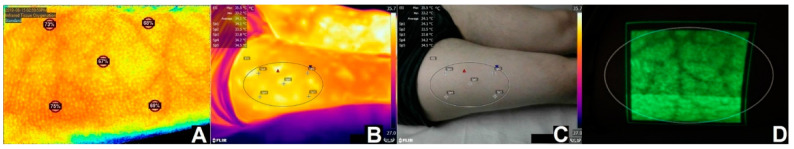
Comparison of the images captured by all devices used in the study at t0; elliptic areas represent the location of topical negative pressure (TNP). (**A**) Image captured by near-infrared spectroscopy. (**B**,**C**) Images captured by thermography, in representative color spectrum and original image. (**D**) Live image created by vein illuminator, captured with digital camera. The white elliptic area represents the location of TNP.

There are numerous studies researching the effects of topical negative pressure application on tissue, and different measurement methods have been used [4,7,18]. Multiple studies used invasive and noninvasive methods such as Doppler imaging, tissue photo spectrometry, white light spectroscopy, or a combination of multiple techniques, including intravital microscopy [8,19,20,21,22]. Our aim was to utilize new and noninvasive methods such as near-infrared spectroscopy and thermography and to evaluate the usefulness of a vein illuminator to improve the knowledge of proposed perfusion changes on superficial tissue (Figure 9). Moreover, we aimed to enhance the understanding of the viability of these new methods to objectify oxygen saturation, skin temperature changes, and potential changes in the venous outflow patterns. With the variations of the pressures of TNPA and adding new measuring tools such as the vein illuminator, we aimed to find out if multi-imaging with the combined tools produces any additional valuable information, in comparison to earlier measurements with different cycles and fewer modalities [16]. It remains hitherto unclear at what external pressure value skin circulation is definitely compromised or supported. Timmers et al. described in a prospective, randomized study the response of cutaneous blood flow (CBF) in healthy intact forearm skin to varying topical negative pressures and different foam types. When they applied a continuous negative pressure within the range of −25 to −500 mmHg and measured skin blood flow with noninvasive laser Doppler probes incorporated into the foam, they observed a significant increase in cutaneous blood flow (CBF) with both foams up to negative pressures of −300 mmHg, with an over 5-fold increase [20].

In line with the studies by Müller-Seubert et al., the initial increase of oxygen saturation directly following the removal of TNPA (control group: −125 mmHg) showed a similar increase, even though the drop of oxygen saturation along the course was higher in this study [16]. Previous studies used high negative pressure points such as −400 mmHg and −500 mmHg to find the critical value where the tissue is under too much pressure so that skin blood flow starts to decrease [19,20]. We have observed increasing values for oxygen saturation for the group with −175 mmHg compared to the control group (−125 mmHg) and did not reach the critical value at which perfusion starts to decrease. Interestingly, for Group 3 (−175 mmHg), we observed a 0.38% difference in oxygen saturation compared to the control group (−125 mmHg) even though we increased the pressure by 40%.

**Figure 10:**
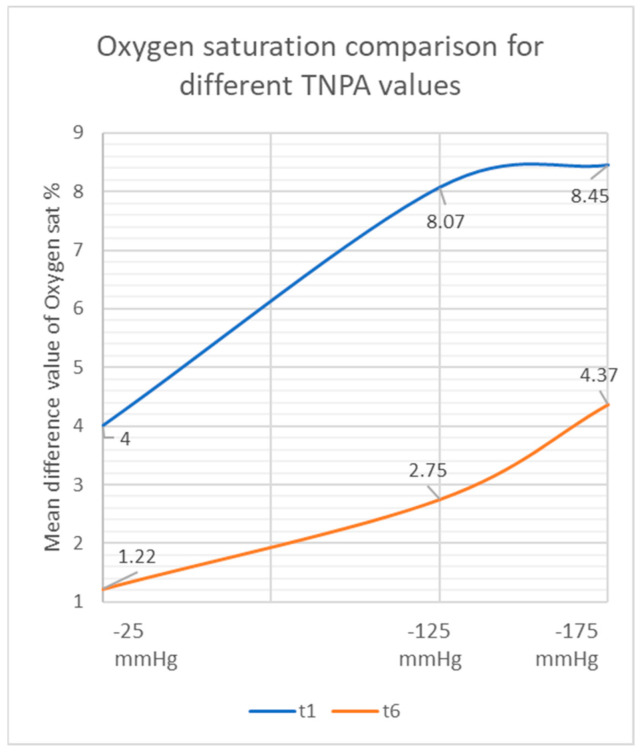
Correlation between the means resulting from different TNP values at t1 and t6.

At the 30 min mark after TNPA removal, Group 3 (−175 mmHg) still presented a higher level of oxygen saturation compared to the control group (−125 mmHg). However, at the same time point, the values of the control group showed higher levels than those of Group 2 (−25 mmHg). Higher levels of negative pressure up to −175 mmHg resulted in higher levels of tissue oxygen both directly after TNPA removal as well as during the progression leading up to 30 min following removal of TNPA (Figure 10). This could be explained by the effect of the higher negative pressures that might help sustain the increased oxygen saturation. By this, the blood flow seems to be enhanced for a longer period compared to lower levels. Concerning the evaluation of tissue oxygen saturation, a clear trend was seen for increased values in higher negative pressure levels. Interestingly, the differences in higher levels exceeding −125 mmHg diminished. This could be interpreted that, at those pressure levels, it comes to a point of no progress or even might reach a turning point. Results of Group 1 (0 mmHg) confirm the assumption that only applying TNPA dressing without a negative pressure does not contribute to tissue oxygen saturation in a meaningful way. For the initial period directly following TNPA removal, a slight persisting increase in oxygen saturation levels was observed in the TNPA groups before values started to decrease.

This is in line with the results by Sogorski et al. in 2018. They found the tissue oxygen saturation increased between TNPA sessions and following the removal of TNPA for a short period of time [21]. The effects of TNPA on the vessel diameter of either veins or arteries in the capillary area remain unclear. Venous vessels might occlude, whereas an arterial vessel can stand a certain level of applied pressure. This might lead to changes in the oxygen saturation and in the blood flow. Furthermore, reactive hyperemia after the removal of TNPA might add to the blood flow alterations as previously discussed.

Concerning the thermography analysis, we found similar developments to those of the oxygen saturation for the initial phase, with an increase of temperature in all groups. Regardless of the negative pressure levels used, the skin temperature showed minimal differences at this point. As for the oxygen saturation values, the relative drop of skin temperature after TNPA removal depended on the pressure level initially set and revealed a higher decrease in the lower pressure levels. Hence, there is a prolonged positive effect on tissue perfusion due to TNPA. It is not able to sufficiently explain the high increase of the skin temperature in Group 1 (0 mmHg), as this might potentially be due to the aforementioned effects on the venous and arterial capillaries. This difference could be due to a lack of depression of veins as a result of no pressure combined with the physiological effect of warming under the dressing causing veins to enlarge, therefore resulting in higher temperature compared to those during TNPA. Due to a potential depression of certain vessels, TNPA could prevent the tissue from warming up over a certain level [21,23,24]; however, we could observe a high drop of skin temperature in Group 1 (0 mmHg) and Group 2 (−25 mmHg) compared to the other groups, underlining the prolonged effects of TNPA.

From generally known physiological effects—as seen in exsanguination procedures in extremity surgery for instance—post occlusive reactive hyperemia effects could explain why the tissue remained at a higher temperature level under higher pressure. In an animal model, when the skin was resected and intravital microscopy was applied, Ichioka et al. found that applying a negative pressure of −125 mmHg significantly increased wound bed blood flow immediately after pressure application, and this was maintained for 1 min after the pressure was released. Whereas, in the −500 mmHg group, the blood flow decreased with time and reached a statistically significant level 5 min after the application of the pressure. This finding is of interest but is not directly applicable to our human direct skin blood flow measurements [22].

One of the aims of our study was to evaluate any additional information that could potentially be gained using a vein illuminator to find out if referable changes in veins can be detected in the focus area. We could see a trend by macroscopic observation; however, due to technical limitations of the device with no built-in clear objective documentation system, we had to rely on the observation of the images we captured macroscopically and subjectively. These observations did not yield additional significant information regarding the exact vein diameters or other structural changes in the venous flow pattern. Nevertheless, it contested that the venous flow pattern remained stable throughout the observation time and was not occluded or significantly altered.

The data of this study are of relevance as, for example, the duration of TNP application over closed incisions has been derived from clinical experiences with the vacuum wound therapy only, while exact data of skin perfusion have been limited so far. From our data, it can be drawn that future studies could aim at a longer time period after the removal of TNPA to see how much time passes for each group to reach baseline and if TNPA for several days shows prolonged effects for an even longer period.

The results are not exactly transferable to an open wound situation. The test should be repeated on open wounds, rather than on healthy participants and intact skin, with TNPA treatment or appropriate animal models to better evaluate the measuring devices. Therefore, we can only evaluate the superficial effects of TNPA. Extended research can better evaluate how useful the devices are for clinical use, possibly showing the effects TNPA has on deeper tissues. Repeating the test with more participants and with a wider range of negative pressures will provide more data about the usefulness of the new devices. Although authors have investigated different pressure settings during TNPA, the basic knowledge relies on the fundamental data initially described by Morykwas et al. [25]. These data were gained from full-thickness wounds on pigs and therefore are not 100% transferable to human intact skin circulation. Studies on wound edge perfusion are also rather scarce and are not exactly comparable to the settings used in our study [9,26].

Studies such as those presented might therefore help to better determine the effects of TNPA on skin microcirculation under various pressures and cycles, albeit the ideal pressure remains yet to be found. For our control group, we chose −125 mmHg negative pressure in accordance with the standard clinical practice and due to it being described by Morykwas et al. as the ideal pressure to induce granulation tissue in full-thickness wounds [19]. To see the effects of the dressing itself, we included Group 1 with only TNPA dressing. We chose −25 mmHg for group 2, as it is the lowest negative pressure possible with the TNPA device we used, to have a baseline for measured values. With the aim of finding the possible ideal value, in group 3, we went slightly above the accepted pressure and used −175 mmHg to see if we could observe a significant increase with a small change. Other studies dealing with the effect of TNPA in different indications and fields proved an effect on peristernal perfusion following sternotomy after harvesting the mammary artery [10]. Degloving injuries were successfully recovered with TNPA, and also facial nerve function preservation has been described to be optimized by it [27,28]. Improved blood circulation in the small intestinal wall was observed by Lindstedt et al. [29]. Local blood flow changes have been successfully utilized to create a prevascularized site for cell transplantation in rats and have been suggested to be a promising method to create a prevascularized site to achieve better results in xenogeneic cell transplantation [30].

## 5. Conclusions

Our investigations underline the efficacy of near-infrared imaging as an appropriate tool to determine changes in microcirculatory skin blood flow patterns under various topical negative pressures delivered via an open cell polyurethan foam. In addition, changes in thermography readings parallel to the values of negative pressures were detectable in the course after TNPA removal. The addition of a new device with near-infrared imaging that was originally developed to visualize venous vessels for the purpose of venipuncture shows a permanent venous blood flow pattern. Despite the initial hypothesis of an inverse relation between pressure exertion and blood flow, we noted that in this study higher levels of negative pressure resulted in higher levels of tissue oxygenation and skin temperature at the end point of 30 min following TNPA. This underlines the potential positive effects of TNPA on tissue perfusion on intact skin or over closed incisions as has been postulated previously from clinical observations.

The results that are presented underline the efficacy of near-infrared imaging and thermography as appropriate tools to determine the changes in microcirculatory blood flow patterns of the skin under various negative pressure levels used during TNPA. The new device used for venous blood analysis showed a permanent venous blood flow pattern and needs further evaluation. For increasing pressure levels, we were able to support the hypothesis that there exists a positive influence of negative pressure wound therapy on skin perfusion. Especially for clinical applications of TNPA on skin, this could influence therapeutic regimes with the need for blood flow alterations. Future research is needed to assess the transferability of the presented results to human wounds.

## Figures and Tables

**Table 1:** Overview of study groups: distribution of participants and TNP values for corresponding groups.

Group	Participant No (n)	(n) Male	(n) Female	TNPA Pressure
Control	24	16	8	−125 mmHg
Group 1	9	6	3	0 mmHg
Group 2	7	5	2	−25 mmHg
Group 3	8	5	3	−175 mmHg

**Table 2:** Overview of the oxygen saturation percentage mean values measured by the near-infrared spectroscopy at t0, t1, t2, and t6. Relative changes in the oxygen saturation between the main time points, negative values representing a decrease in values.

Group	n	t0 Average	t1 Average	t2 Average	t6 Average	Δ t0−t1	Δ t0−t6	Δ t1−t6	t0–t1 Relative Increase	t0–t1 Relative Increase
control	24	63.75%	71.82%	72.24%	66.50%	8.07%	2.75%	−5.32%	12.65%	65.92%
1	9	65.22%	67.77%	68.26%	68.77%	2.55%	3.55%	1.00%	2.55%	39.21%
2	7	64.40%	68.40%	69.22%	65.62%	4.00%	1.12%	−2.78%	6.21%	69.50%
3	8	61.45%	69.90%	70.30%	65.82%	8.45%	4.37%	−4.08%	13.75%	48.28%

**Table 3:** Overview of tissue temperature mean values at t0, t1, and t6, measured by thermography in Celsius degrees. Differences in tissue temperature between the main time points, with negative values representing a decrease in values.

Group	n	t0 Average	t1 Average	t6 Average	Δ t0−t1	Δ t0−t6	Δ t1−t6
control	24	30.60	32.27	31.23	1.67	0.63	−1.04
1	9	30.04	32.75	31.22	2.71	1.18	−1.53
2	7	31.82	33.40	31.84	1.58	0.02	−1.56
3	8	31.55	33.23	32.75	1.68	1.20	−0.48

## Data Availability

The data presented in this study are openly available in FigShare at https://doi.org/10.6084/m9.figshare.20517945.v1.

## References

[1] Horch R.E., Gerngross H., Lang W., Mauckner P., Nord D., Peter R.U., Vogt P.M., Wetzel-Roth W., Willy C. (2005). Indications and safety aspects of vacuum-assisted wound closure. MMW Fortschr. Med..

[2] Hunter J.E., Teot L., Horch R.E., Banwell P.E. (2007). Evidence-based medicine: Vacuum-assisted closure in wound care management. Int. Wound J..

[3] Labanaris A.P., Polykandriotis E., Horch R.E. (2009). The effect of vacuum-assisted closure on lymph vessels in chronic wounds. J. Plast. Reconstr. Aesthet. Surg..

[4] Horch R.E., Ludolph I., Müller-Seubert W., Zetzmann K., Hauck T., Arkudas A., Geierlehner A. (2020). Topical negative-pressure wound therapy: Emerging devices and techniques. Expert. Rev. Med. Devices.

[5] Horch R.E., Braumann C., Dissemond J., Lehner B., Hirche C., Woeste G., Wetzel-Roth W., Willy C. (2018). Use of Negative Pressure Wound Therapy with Instillation and Dwell Time for Wound Treatment—Results of an Expert Consensus Conference. Zentralblatt Chir..

[6] Renno I., Boos A.M., Horch R.E., Ludolph I. (2019). Changes of perfusion patterns of surgical wounds under application of closed incision negative pressure wound therapy in postbariatric patients1. Clin. Hemorheol. Microcirc..

[7] Horch R.E. (2015). Incisional negative pressure wound therapy for high-risk wounds. J. Wound Care.

[8] Muenchow S., Horch R.E., Dragu A. (2019). Effects of topical negative pressure therapy on perfusion and microcirculation of human skin. Clin. Hemorheol. Microcirc..

[9] Malmsjö M., Ingemansson R., Martin R., Huddleston E. (2009). Wound edge microvascular blood flow: Effects of negative pressure wound therapy using gauze or polyurethane foam. Ann. Plast Surg..

[10] Petzina R., Gustafsson L., Mokhtari A., Ingemansson R., Malmsjö M. (2006). Effect of vacuum-assisted closure on blood flow in the peristernal thoracic wall after internal mammary artery harvesting. Eur. J. Cardiothorac. Surg..

[11] Wackenfors A., Sjögren J., Gustafsson R., Algotsson L., Ingemansson R., Malmsjö M. (2004). Effects of vacuum-assisted closure therapy on inguinal wound edge microvascular blood flow. Wound Repair. Regen..

[12] Lou D., Pang Q., Pei X., Dong S., Li S., Tan W.-Q., Ma L. (2020). Flexible wound healing system for pro-regeneration, temperature monitoring and infection early warning. Biosens. Bioelectron..

[13] Power G., Moore Z., O’Connor T. (2017). Measurement of pH, exudate composition and temperature in wound healing: A systematic review. J. Wound Care.

[14] Castilla D.M., Liu Z.J., Velazquez O.C. (2012). Oxygen: Implications for Wound Healing. Adv. Wound Care.

[15] Yip W.L. (2015). Influence of oxygen on wound healing. Int. Wound J..

[16] Muller-Seubert W., Roth S., Hauck T., Arkudas A., Horch R.E., Ludolph I. (2021). Novel imaging methods reveal positive impact of topical negative pressure application on tissue perfusion in an in vivo skin model. Int. Wound J..

[17] Müller-Seubert W., Ostermaier P., Horch R.E., Distel L., Frey B., Cai A., Arkudas A. (2022). Intra- and Early Postoperative Evaluation of Malperfused Areas in an Irradiated Random Pattern Skin Flap Model Using Indocyanine Green Angiography and Near-Infrared Reflectance-Based Imaging and Infrared Thermography. J. Pers. Med..

[18] Geierlehner A., Horch R.E., Muller-Seubert W., Arkudas A., Ludolph I. (2020). Limb salvage procedure in immunocompromised patients with therapy-resistant leg ulcers—The value of ultra-radical debridement and instillation negative-pressure wound therapy. Int. Wound J..

[19] Morykwas M.J., Argenta L.C., Shelton-Brown E.I., McGuirt W. (1997). Vacuum-assisted closure: A new method for wound control and treatment: Animal studies and basic foundation. Ann. Plast. Surg..

[20] Timmers M.S., Le Cessie S., Banwell P., Jukema G.N. (2005). The effects of varying degrees of pressure delivered by negative-pressure wound therapy on skin perfusion. Ann. Plast. Surg..

[21] Sogorski A., Lehnhardt M., Goertz O., Harati K., Kapalschinski N., Hirsch T., Daigeler A., Kolbenschlag J. (2018). Improvement of local microcirculation through intermittent negative pressure wound therapy (NPWT). J. Tissue Viability.

[22] Ichioka S., Watanabe H., Sekiya N., Shibata M., Nakatsuka T. (2008). A technique to visualize wound bed microcirculation and the acute effect of negative pressure. Wound Repair. Regen..

[23] Kairinos N., Solomons M., Hudson D.A. (2009). Negative-pressure wound therapy I: The paradox of negative-pressure wound therapy. Plast. Reconstr. Surg..

[24] Kairinos N., Voogd A.M., Botha P.H., Kotze T., Kahn D., Hudson D.A., Solomons M. (2009). Negative-pressure wound therapy II: Negative-pressure wound therapy and increased perfusion. Just an illusion?. Plast. Reconstr. Surg..

[25] Morykwas M.J., Faler B.J., Pearce D.J., Argenta L.C. (2001). Effects of varying levels of subatmospheric pressure on the rate of granulation tissue formation in experimental wounds in swine. Ann. Plast. Surg..

[26] Malmsjö M., Ingemansson R., Martin R., Huddleston E. (2009). Negative-pressure wound therapy using gauze or open-cell polyurethane foam: Similar early effects on pressure transduction and tissue contraction in an experimental porcine wound model. Wound Repair. Regen..

[27] Andres T., von Lübken F., Friemert B., Achatz G. (2016). Vacuum-Assisted Closure in the Management of Degloving Soft Tissue Injury: A Case Report. J. Foot Ankle Surg..

[28] Linkov G., Cracchiolo J., Fielding A.F., Liu J.C. (2014). Facial nerve function preservation with vacuum-assisted closure. J. Craniofac. Surg..

[29] Lindstedt S., Malmsjö M., Hansson J., Hlebowicz J., Ingemansson R. (2012). Microvascular blood flow changes in the small intestinal wall during conventional negative pressure wound therapy and negative pressure wound therapy using a protective disc over the intestines in laparostomy. Ann. Surg..

[30] Stiegler P., Matzi V., Pierer E., Hauser O., Schaffellner S., Renner H., Greilberger J., Aigner R., Maier A., Lackner C. (2010). Creation of a prevascularized site for cell transplantation in rats. Xenotransplantation.

